# LASSO regression shows histidine and sphingosine 1 phosphate are linked to both sepsis mortality and endothelial damage

**DOI:** 10.1186/s40001-023-01612-7

**Published:** 2024-01-20

**Authors:** Pär I. Johansson, Hanne H. Henriksen, Sigurður T. Karvelsson, Óttar Rolfsson, Martin Schønemann-Lund, Morten H. Bestle, Sarah McGarrity

**Affiliations:** 1 CAG Center for Endotheliomics, Copenhagen University Hospital - Rigshospitalet, Copenhagen, Denmark; 2 Department of Clinical Medicine, University of Copenhagen, Copenhagen, Denmark; 3 Biomedical Center, University of Iceland, Reykjavik, Iceland; 4 Department of Anaesthesiology and Intensive Care, Copenhagen University Hospital - North Zealand, Hillerod, Denmark

## Abstract

Sepsis is a major cause of death worldwide, with a mortality rate that has remained stubbornly high. The current gold standard of risk stratifying sepsis patients provides limited mechanistic insight for therapeutic targeting. An improved ability to predict sepsis mortality and to understand the risk factors would allow better treatment targeting. Sepsis causes metabolic dysregulation in patients; therefore, metabolomics offers a promising tool to study sepsis. It is also known that that in sepsis endothelial cells affecting their function regarding blood clotting and vascular permeability. We integrated metabolomics data from patients admitted to an intensive care unit for sepsis, with commonly collected clinical features of their cases and two measures of endothelial function relevant to blood vessel function, platelet endothelial cell adhesion molecule and soluble thrombomodulin concentrations in plasma. We used least absolute shrinkage and selection operator penalized regression, and pathway enrichment analysis to identify features most able to predict 30-day survival. The features important to sepsis survival include carnitines, and amino acids. Endothelial proteins in plasma also predict 30-day mortality and the levels of these proteins also correlate with a somewhat overlapping set of metabolites. Overall metabolic dysregulation, particularly in endothelial cells, may be a contributory factor to sepsis response. By exploring sepsis metabolomics data in conjunction with clinical features and endothelial proteins we have gained a better understanding of sepsis risk factors.

**Supplementary Information:**

The online version contains supplementary material available at 10.1186/s40001-023-01612-7.

## Introduction

Sepsis is a major cause of death worldwide, accounting for an estimated 11 million deaths in 2017 [1]. A large meta-analysis found that there has been no significant decrease since 2009 [2].

To improve sepsis outcomes consensus definitions have been proposed and updated. The two most recent iterations date from 2016 [3–6] (used in this study) and 2021 [7]. The 2016 guidelines define sepsis as a sequential organ failure assessment (SOFA) score change of > 2, at bedside this can be made using quick (q) SOFA, and septic shock based on hypotension requiring vasopressors and elevated serum lactate. This stratification was primarily designed to guide the treatment plans of sepsis patients. However, it also correlates with the outcome of patients, it shows significant variability in predictive performance between studies [8, 9]. Therefore, further research to identify those patients at most risk of death is necessary to direct therapeutic interventions to improve patient outcomes. Moreover, septic shock diagnosis does not stratify patients in a mechanistic fashion. A more mechanistic stratification could better inform treatment strategies. To facilitate mechanistic patient stratification, it is necessary to define biomarkers with prognostic ability that will also improve understanding of the underlying biology defining different patient risk groups. This may also eventually lead to more targeted treatment options.

Endothelial dysfunction, and changes related to endothelial-linked proteins, including Soluble thrombomodulin (sTM) and Platelet and Endothelial Cell Adhesion Molecule 1 (PECAM), have been linked to the development of multi-organ failure and death in sepsis patients [10–14]. This pro-apoptotic, pro-inflammatory, initially pro-coagulant and pro-adhesive response of vascular endothelial cells, contributes to many of the circulatory and immunological pathologies leading to death in sepsis [15, 16].

Lactate and base excess are predictive of sepsis outcome [17, 18], these indicate metabolic and respiratory dysfunction. However, lactate and base excess primarily indicate changes in central metabolism or patient respiratory function in general and do not offer more detailed insights into other aspects of metabolism. To identify more specific metabolic markers over the past decade an increasing number of metabolomic studies have elucidated the changes seen in sepsis patient metabolite levels [19–23]. However, a consensus about a useful biomarker panel has not yet been reached and there has not been a clear linkage of identified metabolites to functional changes in the pathology of sepsis. This group and others, have previously shown that endothelial cell metabolism is linked to endothelial function after pathological stimulation [24–26], in patients’ plasma metabolites will not only reflect the metabolism of endothelial cells but will likely partly reflect this. Therefore, we proposed that metabolic biomarkers that are linked to sepsis mortality will also be linked to biomarkers of endothelial health. We also believe that this will provide useful directions for future research into the complex mechanisms behind sepsis mortality.

To address the lack of prognostic and mechanistic biomarkers in sepsis we have used a metabolomics data set collected from patients admitted to an intensive care unit (ICU) at a regional hospital who were diagnosed as suffering from either sepsis or septic shock on admission. We have then taken a least absolute shrinkage and selector operator (LASSO) regression approach [27], a form of penalised regression, to identify metabolites most strongly related to sepsis 30-day survival or to the endothelial health biomarkers, PECAM and sTM, and then examined the relationship between the two to identify functionally relevant biomarkers for sepsis survival.

## Methods

### Patient selection

Between November 2017 and September 2018 at the Copenhagen University Hospital North Zealand, in Hillerod Denmark patients admitted to the intensive care unit (ICU) with sepsis were considered for inclusion in this study. The additional inclusion criteria were age ≥ 18, estimated survival above 24 h, and fulfilment of sepsis criteria according to the Surviving Sepsis Guidelines 3 2016 [4]. Exclusion criteria were the existence of a do not resuscitate order and if the expected ICU stay was < 24 h. The patients were included in the study and samples taken at admission to the ICU, all patients, therefore, required intensive care level treatment, most often some sort of vital organ support. All patients in this study had sepsis or septic shock at the time of admission to the ICU, some had been admitted to another ward prior to admission to ICU, while others were admitted from the community. This resulted in a data set of 52 patients. The Ethics Committees in the Capital Region of Denmark approved this study (H-17027963 and Danish Data Protection Agency (I-suite nr.: 04673 and 04674)). Written informed consent was obtained from all subjects. Patients were enrolled after informed consent, from next of kin, if available, and otherwise from the patient´s general practitioner. In patients who regained consciousness, informed consent was obtained as soon as possible thereafter.

### Data collection and electronic health record processing

Blood samples were obtained as soon as possible after an ICU admission that met the criteria described above for enrolment. These samples were used for metabolomics measurements by MSOmics and measurement of endothelial health markers by ELISA. Clinical and biochemical data for this study was obtained from a dedicated study database (REDCap) extracted from the patients’ electronic health records (Sundhedsplatformen, provided by EPIC [28]), data found in supplementary data. Included in this were data about the initial ICU admission, standard biochemical laboratory measurements and information about the patients’ characteristics and health history. Patients were characterised as having sepsis or septic shock according to the 2016 criteria from the sepsis 3 guidelines [4]. The SOFA score—arterial/inspired oxygen pressure ratio, mechanical ventilation, platelet count, cognitive function, bilirubin concentration, creatinine concentration and mean arterial pressure—was also calculated and included in the data set.

### Endothelial biomarker analysis

The soluble biomarkers of endothelial damage sTM (CD141) and PECAM (CD31) were measured by immunoassays according to the manufacturer’s recommendation (Co. KG, Nordhorn, Germany and Diaclone Nordic Biosite, Copenhagen, Denmark & R&D systems). These biomarkers were selected as representing two different aspects of endothelial health, and their significance to the recovery from sepsis as measured by release from mechanical ventilation in a previous study by this group [29]. sTM levels indicates primarily direct damage to the endothelial cells, while PECAM level indicates more specifically disruption of the tight junctions between the endothelial cells.

### Metabolomics mass-spectrometry analysis

Ultra-high-performance liquid chromatography–mass spectrometry (UHPLC–MS) analysis and gas chromatography–mass spectrometry (GCMS) analysis were performed by MSOmics (Vedbæk, Denmark). Peaks were identified and quantified for 63 metabolites (listed in Additional file 1: Data S1, including a list of which metabolites were measured by UHPLC–MS or GCMS, while an untargeted multiple reaction monitoring method was used these 63 metabolites of interest were quantified by comparison to known standards and it was these metabolites that were included in this study). To reduce systematic variations introduced during sample preparation and analysis, MSOmics processed samples in a randomised order. Furthermore, for quality control, a mixed pooled sample, consisting of a small aliquot from each sample, was analysed at regular intervals throughout the sequence.

The UPLC–MS analysis was performed using a slightly modified version of the protocol described by Catalin et al*.* (UPLC/MS Monitoring of Water-Soluble Vitamin Bs in Cell Culture Media in Minutes, Water Application note 2011, 720004042en) [30]. Plasma (90μL), internal standard (10μL) and acetonitrile with 1% formic acid (400μL) were added to the Phree phospholipid removal filters. The filters were centrifuged (10 min at 4 °C and 1000g). The filtrate was diluted (1:1) in 10mM ammonium formate with 0.1% formic acid. Mobile phase A was 10 mM ammonium formate, 0.1% formic acid in water (pH 3.1), mobile phase b was 10 mM ammonium formate, 0.1% formic acid in methanol. A 15-min gradient method, at 300 μL/min was used. Beginning with 100% mobile phase A, reaching 90% mobile phase B after 13 min and ending in 100% mobile phase A. The MS analysis was carried out using a Thermo Scientific Vanquish LC coupled to Thermo Q Exactive HF MS. An electrospray ionization interface was used as ionization source. Analysis was performed in negative and positive ionization mode and the mass spectrometry settings shown in Table 1. Peak areas were extracted using Compound Discoverer 2.0 (Thermo Scientific).

**Table 1:** Tuning settings used for the mass spectrometer for the liquid chromatography mass spectrometry method. HESI (Heated Electrospray Ionization)

Setting	Value
Spray voltage ( +)	3500
Spray voltage (−)	2500
Capillary temperature (+ or + −)	320
Capillary temperature (−)	320
Sheath gas (+ or + −)	47,5
Sheath gas (−)	47,5
Auxiliary gas (+ or + −)	11,25
Auxiliary gas (-)	11,25
Spare gas (+ or + −)	2,25
Spare gas (−)	2,25
Max spray current ( +)	100
Max spray current (−)	100
Probe heater temperature (+ or + −)	412,5
Probe heater temperature (−)	412,5
S-lens RF level	50
Ion source	HESI

GC–MS analysis was carried out by MS-Omics as follows. First 150μL filtrate were dried under nitrogen flow and reconstituted in 37μL ultra-pure water. The reconstituted samples were derivatized with methyl chloroformate using a slightly modified version of the protocol described by Smart et al. [31]. All samples were analysed in a randomized order. Analysis was performed using gas chromatography (7890B, Agilent) coupled with a quadropole mass spectrometry detector (5977B, Agilent). The system was controlled by ChemStation (Agilent). Raw data were converted to netCDF format using Chemstation (Agilent), before the data were imported and processed in Matlab R2018b (Mathworks, Inc.) using the PARADISe software [32].

All quantifications were performed using external calibration rows. Compounds quantified using the GC–MS platform were normalised to alanine-4d, while selected compounds quantified using the UHPLC–MS platform (both positive and negative mode) were normalised using one of five internal standards (Carnitine-d9, Glucose-13C6, Glutamic acid-d5, Phenylalanine-d8, or TMAO-d9). The selection of which internal standard to use (if any) was based on an evaluation of which choice most effectively removed matrix effects in plasma based on spiking experiments.

### Characterisation of sepsis survivors vs non-survivors

Using R the differences between patients with a greater than 30-day survival and less than 30-day survival from ICU admission for sepsis were defined. For the clinical and patient history features including the markers of endothelial dysfunction Fisher’s exact test (discrete variables) and Mann–Whitney *U* test (continuous variables) were performed with the rstatix package [33].

### Data filtering and quality control

The data, including the reported metabolite concentrations, the various clinical features, was processed using R. An initial assessment of the data led to the removal of variables that were closely linked, for example, date of birth was removed but age retained. This produced a data set with 52 patients and 96 features (metabolites, endothelial markers and clinical characteristics see Additional file 1) plus the 30-day survival outcomes for each patient.

Features that were missing measurements in more than 10% of patients were identified. A total of 14 features were found to contain greater than 10% missing values. Of these 13 were metabolites, and removed, from all subsequent analyses. BMI was retained for the initial characterisation of patients but not included in later analyses.

Many measurements of hypoxanthine were below 0, suggesting that the quantification was poor. Therefore, this metabolite was also excluded from further analysis.

Next it was determined that no patients lacked greater than 10% of features, so all were retained.

For remaining metabolites missing values were imputed using the missRanger algorithm from the Ranger R package [34], then the data were log and pareto scaled.

### Selection and assessment of the predictive power of various features for 30-day survival.

To assess the utility of various features as a predictive tool for sepsis survival receiver operator curve (ROC) characteristics were used via the pROC package [35] in R. We used the lm function in R to fit models accounting for either septic shock diagnosis [4] or PECAM and sTM concentration combined [10–14]. All observations were used for fitting in a bootstrap approach due to the small sample size. The pROC package was then used to establish the area under the curve (AUC) for ROC curves for these models.

$$Cost = \frac{1}{2N}\mathop \sum \limits_{{\left( {i = 1} \right)}}^{N} \left( {y_{i} - \mathop \sum \limits_{{\left( {j = 1} \right)}}^{p} \left[\kern-0.15em\left[ {w_{j} {*}x_{i} j} \right]\kern-0.15em\right]} \right)^{2} + \lambda \mathop \sum \limits_{{\left( {j = 1} \right)}}^{p} \left[\kern-0.15em\left[ {v_{j} {*}\left| {w_{j} } \right|} \right]\kern-0.15em\right]$$

The glmnet package [36] was used to implement a LASSO regression. LASSO regression requires the selection of the optimal value for the hyperparameter lambda (λ) by minimisation of the cost function, Eq. 1. This optimisation was performed by nested k-fold cross-validation. The data set was partitioned to form 5 groups of which 4 are used as a subset to regress all metabolite parameters and the significantly altered clinical variables on a y variable of interest (either death within 30 days of admission, sTM or PECAM). From these regressions an optimal lambda is identified using an internal cross-validation that minimizes the cost function to get the best fitting regression line. Where N is the number of patients, p is the number of predictors, λ is the lambda hyperparameter, wj is the coefficient for predictor j and v is the penalty vector, which tunes the amount of penalization of each predictor. The penalty vector values for all predictors were 1. The median of the optimal lambda values from the five partitions was identified. This fivefold cross-validation process was repeated 500 times to build a sparse linear regression model, consisting of the metabolites associated with each of the variables of interest. Details of the repeated cross-validations are shown in Additional file 2.

For the LASSO regression, the glmnet package was used. LASSO regression extends regular linear regression by adding a penalty term that encourages sparsity in the model, leading to potential feature selection benefits (Eq. 1). Regular linear regression, on the other hand, does not include any regularization or penalty terms. The regularization parameter λ needs to be selected carefully to balance accuracy (good data-fitting) and interpretability (sparsity) of the model.

Due to a small sample size, a bootstrap resampling procedure was used to identify an optimal λ. For each bootstrap sample, fivefold cross validation was employed to assess the performance of the LASSO regression model across a range of λ values. The goal was to identify the optimal λ that minimizes the cross-validated mean squared error. A median λ value from the 500 bootstrap samples was determined (Additional file 2), providing robustness to the model selection process. This median λ value was then used in a final LASSO regression model, where all the original metabolite measurements were fitted to predict the response variable of interest (i.e., sTM, PECAM and 30-day survival rate).

### Investigation of the mechanisms behind poor outcomes in sepsis

First, the links between the markers of endothelial dysfunction and metabolism were investigated by performing LASSO regression as above but with the model being fitted to PECAM or sTM concentration and instead of survival.

Second, the areas of metabolism linked to survival and endothelial dysfunction were investigated using over-representation analysis run via the deCoupleR [37] package and the KEGG pathways [38] section of the chemical/chemical interaction network from the Stitch database (downloaded May 2022) [39] using the size of the coefficients found by LASSO regression analysis for 30-day survival, PECAM and sTM concentrations.

## Results

### Analysis of patient characteristics and clinical features on admission show few differences between survivors and non-survivors of sepsis at 30 days

Basic demographic parameters and PECAM and sTM, endothelial health indicators, were compared between survivors and non-survivors. Of a total of 52 patients in this study 19 died within 30 days of ICU admission for sepsis. The mean age of patients in this study was 71, not significantly different between survivors and non-survivors, see Table 2. The sex of patients was evenly split between males and females (25 males) and the 30-day survival rate was the same for both, Table 2. BMI was lower in patients who died within 30 days of ICU admission, Table 2.

**Table 2:** *Patient Characteristics comparing those with 30-day survival vs 30-day all-cause mortality from ICU sepsis admission, P values from Fishers Exact test (discrete variables) or Mann–Whitney U test (continuous variables) (F* = *Female, M* = *Male, BMI* = *Body Mass Index, SOFA* = *Sequential Organ Failure assessment, PECAM* = *Platelet Endothelial Cell Adhesion Molecule)*

		Status		
Category	Feature	Survivors [Counts/Median (Lower and Upper Quartiles)]	Non-survivors [counts/median (lower and upper quartiles)]	*p* value
*Demographic Information*	Sex	F-17, M-16	F-10, M-9	1.000
	Reported smoking status	Ex-smoker-12, no-11, yes-7, missing-3	Ex-smoker-10, No-6, yes-2, missing-1	0.679
	Age (years)	71 (66–79)	72 (68.5–78.5)	0.879
	***BMI***	***31.419 (25.703–33.951)***	***24.412 (19.891–27.244)***	***0.036***
*Endothelial Feature*	***PECAM (ng/mL)***	***10.8 (9.33–13.3)***	***15.4 (12.25–19)***	***0.005***
	***Soluble Thrombomodulin (ng/mL)***	***13.21 (10.42–18.73)***	***20.17 (14.675–24.21)***	***0.006***
*Measurement at ICU Admission*	***Base excess (mM)***	***1.8 (1.2–2.6)***	***2.8 (1.95–3.775)***	***0.020***
	Lactate (mM)	7 (6.3–7.8)	6.5 (5.8–8.5)	0.894
	***SOFA***	***8 (7–9)***	***13 (10–14)***	***0.001***
	Met the criteria for septic shock	Septicshock-16, Sepsis-17	SepticShock-12, Sepsis-7	0.391

We considered the effects of patient health history and the reason for admission secondary to sepsis. Only an existing history of liver cirrhosis or diabetes were significantly over-represented among non-survivors (*p* = 0.004 and 0.011, respectively), see Supplement 2a. Liver disease as an admission reason was also over-represented among non-survivors (*p* = 0.044) (see Additional file 3b).

Patients who died within 30 days of the sepsis diagnosis had a higher base excess, SOFA score, plasma PECAM concentration, and plasma sTM concentration than those who survived (*p* = 0.02, 0, 0 and 0.01, respectively), see Table 2. However, there were no differences in survival by vasopressor treatment, mechanical ventilation use or those septic shock diagnosis, see Additional file 3a.

### Current predictors of survival in sepsis perform similar to markers of endothelial health

We examined three previously reported methods of predicting 30-day all-cause mortality, septic shock diagnosis, SOFA score and a combination of two previously reported markers of endothelial health and sepsis outcome, PECAM and sTM.

**Fig. 1:**
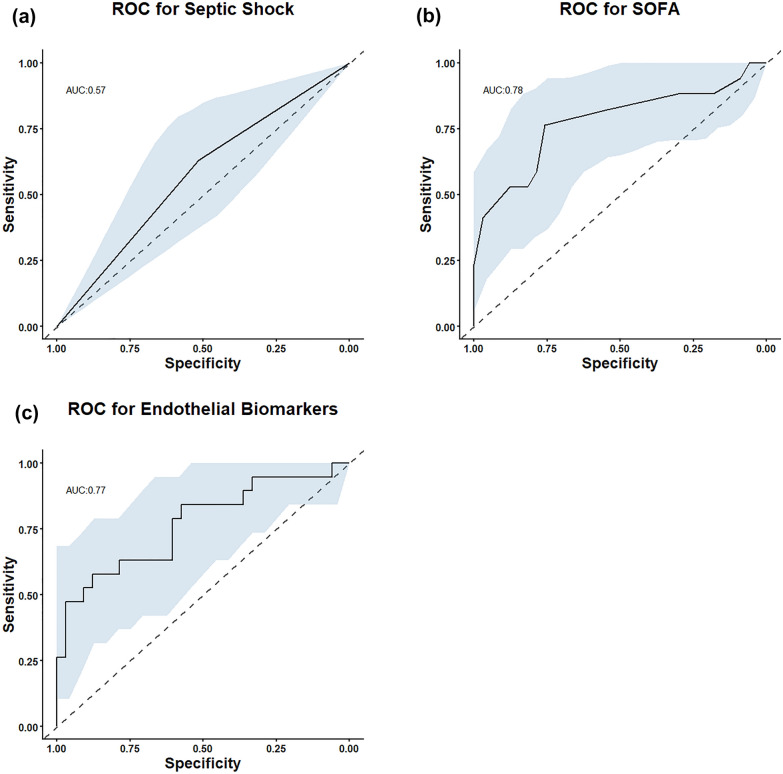
ROC curves assessing the predictive power of **a** Septic shock diagnosis. **b** SOFA score, or **c** combination of PECAM and sTM, indicators of vascular endothelial health

In this study a septic shock diagnosis was only slightly more able than chance to predict 30-day mortality, area under the curve (AUC) of 0.57 (0.432–0.714), see Fig. 1a.

The full SOFA score showed an AUC of 0.77 (95% CI (0.624–0.93)) for 30-day mortality, see Fig. 1b.

The combination of both endothelial health indicators, PECAM and sTM, was able to predict 30-day mortality well with an AUC of 0.77 (95% CI 0.626–0.911), see Fig. 1c. This suggests that endothelial health was a good indicator of sepsis survival, in the study population.

### A LASSO regression model suggests metabolites as predictors of sepsis survival

To identify the metabolites that would be the best biomarkers for sepsis survival we used penalised regression. We then assessed the ability of this model and the most important metabolites to predict sepsis survival. LASSO regression models show 30-day survival as being best predicted by 15 metabolites. Nine metabolites that are positively correlated with 30-day mortality and six which are negatively correlated with 30-day mortality, see Table 3. Many of these metabolites are amino acids or carnitines.

**Table 3:** Results of LASSO regression as associated with 30-day survival, sTM and PECAM

Correlation	Metabolite	Coefficient
*Non-survival*	Glycine	− 0.011
	Isoleucine	− 0.124
	Asparagine	0.035
	Glutamate	− 0.012
	Tyrosine	0.090
	Pyruvate	− 0.065
	Fumarate	0.083
	Oxoproline	0.101
	Butyrylcarnitine	0.028
	Docosahexanoate	− 0.036
	Glutamine	0.029
	Histidine	0.111
	Myristoylcarnitine	0.055
	Sphingosine 1 phosphate	− 0.049
	Urate	0.004
*sTM*	Glutamate	− 1.327
	Ornithine	1.064
	Tryptophan	− 0.650
	Cystine	− 0.278
	Fumarate	1.221
	Acetylcarnitine	0.845
	Eicosapentanoate	− 0.102
	Histidine	1.296
	Linoleate	− 0.896
	Kynurenine	0.865
	Myristoylcarnitine	0.606
	Octanylcarnitine	1.409
	Sphingosine 1 phosphate	− 1.729
	Trimethyl N oxide	0.144
	Urate	0.548
*PECAM*	Glycine	− 1.728
	Valine	− 1.222
	Isoleucine	− 1.083
	Threonine	3.463
	Glutamate	0.978
	Phenylalanine	− 0.104
	Tryptophan	− 0.983
	Aspartate	0.887
	Pyruvate	− 0.435
	Succinate	− 0.430
	Arachidonate	0.671
	Arginine	0.732
	Docosahexanoate	− 0.897
	Gamma Linolenicate A	− 0.605
	Glucose	− 0.610
	Glutamine	0.206
	Histidine	1.869
	Linolenate	− 0.212
	Myristoylcarnitine	− 0.379
	Propionylcarnitine	0.107
	Sphingosine 1 phosphate	− 0.865
	Trimethyl N oxide	0.363
	Urate	1.832

**Fig. 2:**
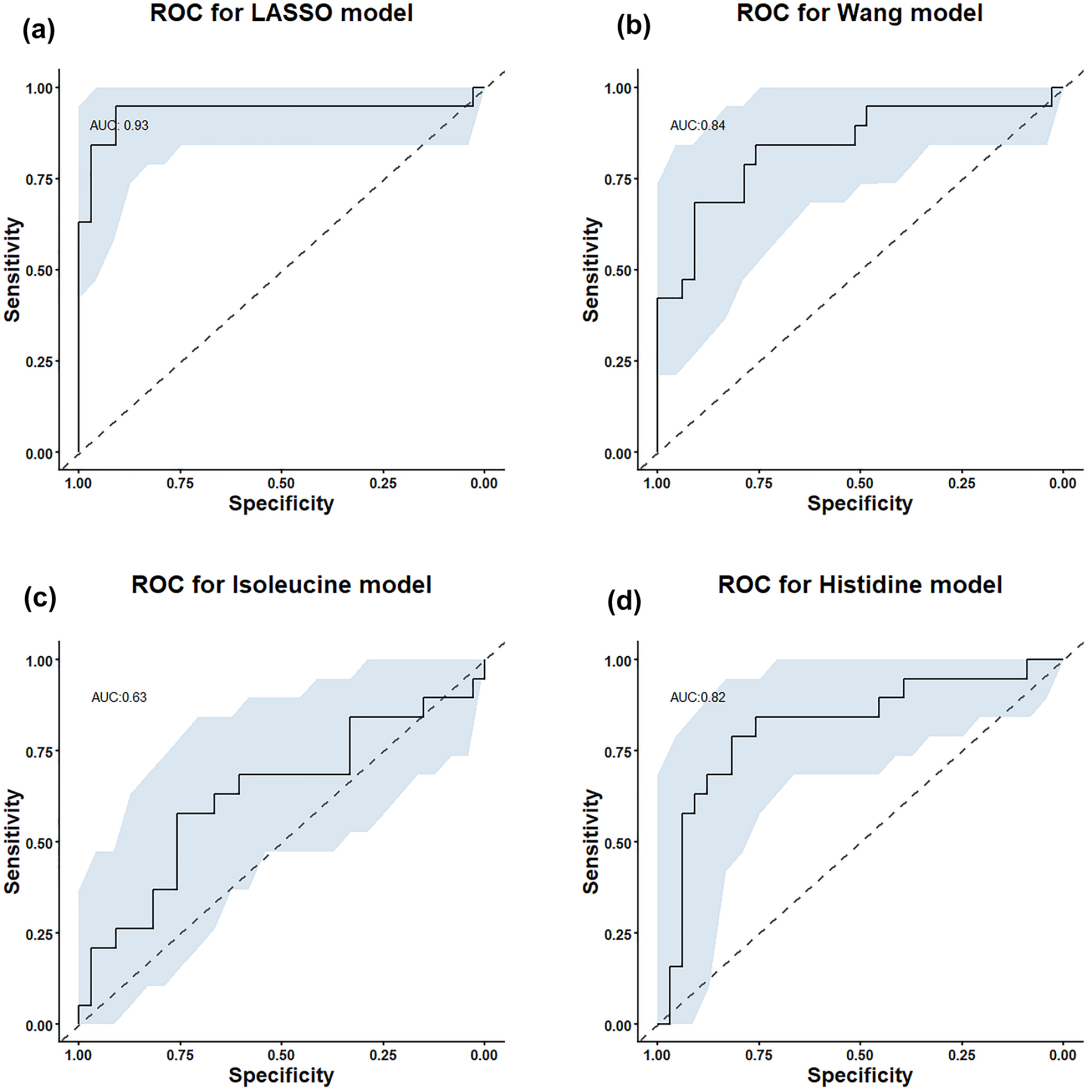
ROC curves assessing the predictive power of various metabolites. **a** In the data set from this paper and the LASSO regression selected metabolites. **b** Consensus model from Wang et al., with as many metabolites as possible included **c** isoleucine and **d** histidine all predicting 30-day survival in sepsis patients

The ROC curve for this model shows that it predicts 30-day survival very well with an AUC of 0.94 (95% CI 0.841-1), see Fig. 2a. Overlapping 95% confidence intervals of the AUC of these two models show that this was an equally good model as that Wang et al. [40] (including isoleucine, acetylcarnitine, lactate and pyruvate) applied to our data set, see Fig. 2b.

Models for each of the two metabolites with the largest coefficients in the LASSO model were tested. Isoleucine showed a moderately good predictive ability alone, AUC of 0.628 [95% CI (0.459–0.798)] for 30-day mortality, see Fig. 2c. Histidine was a better predictor, AUC of 0.818 [95% CI (0.687–0.949)] for 30-day mortality, see Fig. 2d.

We found that a group of metabolic markers, including isoleucine and histidine, were able to predict 30-day survival very well.

### Endothelial health markers and sepsis survival are best modelled by overlapping sets of metabolites

**Fig. 3:**
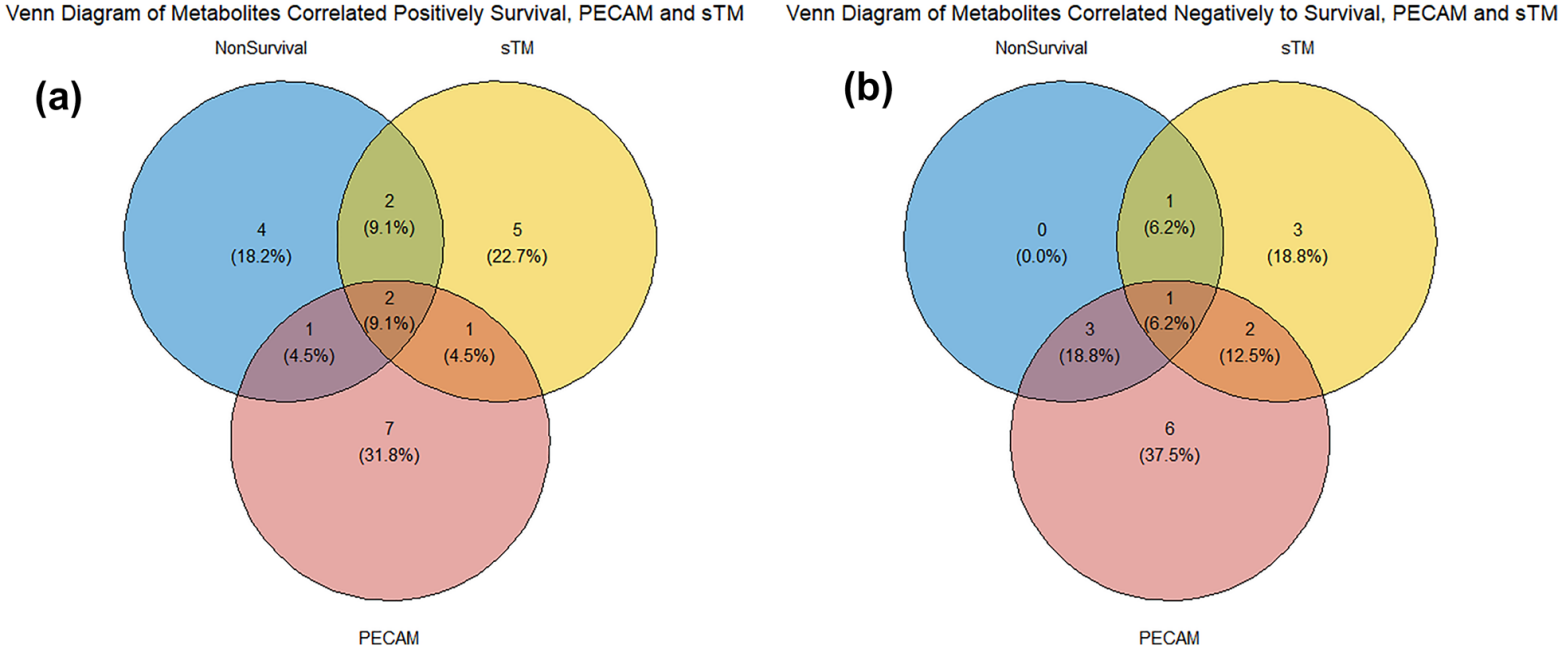
Venn diagrams showing the proportion of metabolites shared between regression models of mortality, PECAM and sTM. **a** Positive correlation. **b** Negative correlation

We next used penalised regression to identify metabolites that correlate with sTM and PECAM, markers of endothelial health. We then identified the overlap between the three sets of metabolites. LASSO regression shows that plasma PECAM levels are positively correlated with 10 metabolites, and negatively correlated with 13 metabolites, see Table 3 and Fig. 3a and b. A similar model for sTM shows a positive correlation with 9 metabolites and, and negatively with 6 metabolites, see Table 3 and Fig. 3a and b.

Several metabolites correlate with sTM, PECAM and 30-day mortality. Histidine, and urate are a both positively associated with all three traits, while sphingosine 1 phosphate is negatively associated with all three traits, see Additional files 3 and 4. Interestingly there are no metabolites that are positively related to both PECAM and sTM but not to mortality. Furthermore, histidine is one metabolite that is most strongly associated with 30-day sepsis mortality.

### Enrichment analysis shows the importance of central carbon and amino acid metabolism to sepsis response

**Fig. 4:**
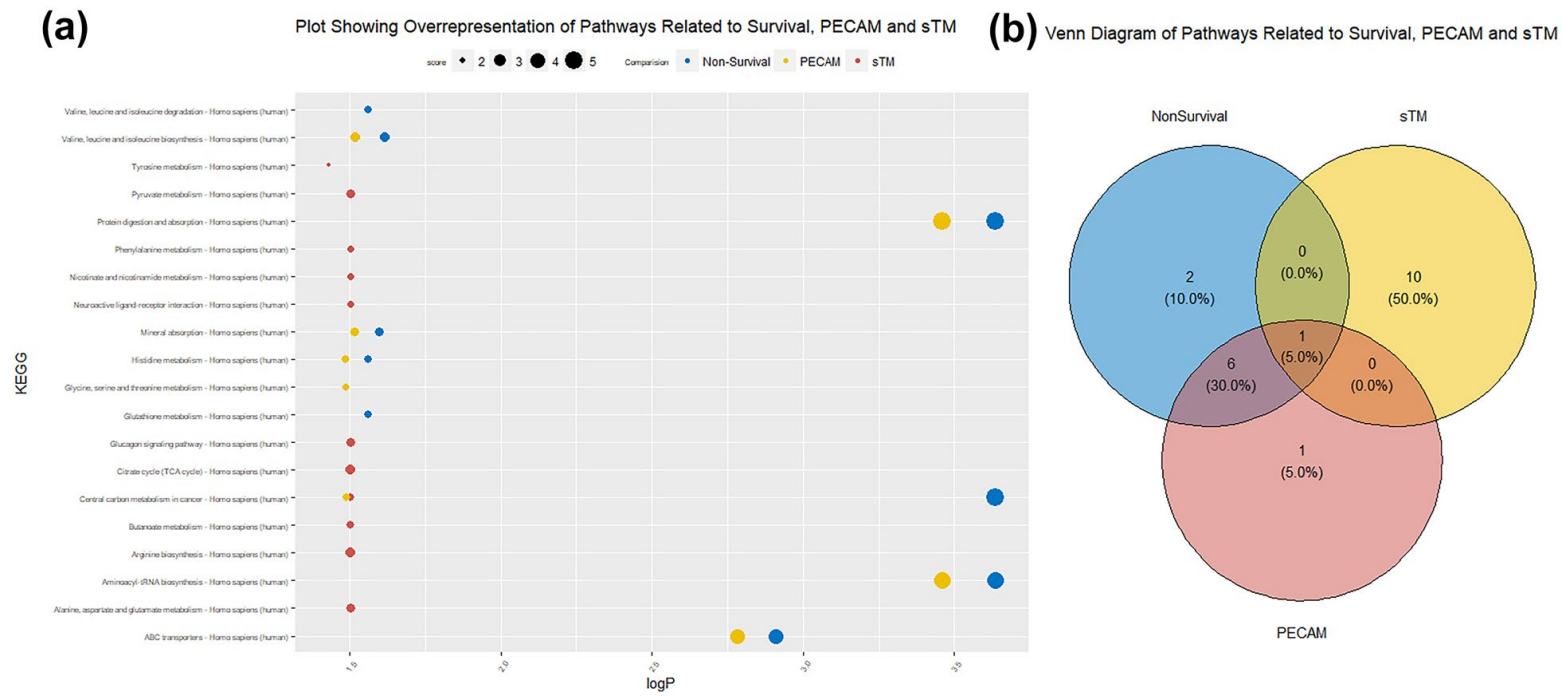
A figure showing significantly overrepresented KEGG pathways in metabolites highlighted by LASSO regression defining **a** sepsis survival, sTM and plasma PECAM by a plot of points located based on the ORA—log of FDR adjusted *p* value and size of the point defined by the ORA score and **b** a Venn diagram showing the shared and unique pathways from part **a**

Over-representation analysis (ORA) was performed to compare the sets of metabolites identified by LASSO regression as being linked to 30-day mortality, plasma sTM concentration or plasma PECAM concentration, to the KEGG pathways [38] found in the Stitch database [39], significant results shown in Table 4 and Fig. 4a.

**Table 4:** Results of ORA showing the over-represented pathways for metabolites identified by LASSO regression as associated with 30-day survival, sTM and PECAM

Comparison	KEGG	ORA Score	Adjusted *p* value
*Non-survival*	ABC transporters—Homo sapiens (human)	3.850	0.001
	Aminoacyl-tRNA biosynthesis—Homo sapiens (human)	4.702	0.000
	Central carbon metabolism in cancer—Homo sapiens (human)	5.001	0.000
	Glutathione metabolism—Homo sapiens (human)	2.245	0.027
	Histidine metabolism—Homo sapiens (human)	2.153	0.027
	Mineral absorption—Homo sapiens (human)	2.362	0.025
	Protein digestion and absorption—Homo sapiens (human)	4.791	0.000
	Valine, leucine and isoleucine biosynthesis—Homo sapiens (human)	2.463	0.024
	Valine, leucine and isoleucine degradation—Homo sapiens (human)	2.202	0.027
*sTM*	Alanine, aspartate and glutamate metabolism—Homo sapiens (human)	2.377	0.031
	Arginine biosynthesis—Homo sapiens (human)	2.463	0.031
	Butanoate metabolism—Homo sapiens (human)	2.202	0.031
	Central carbon metabolism in cancer—Homo sapiens (human)	2.256	0.031
	Citrate cycle (TCA cycle)—Homo sapiens (human)	2.523	0.031
	Glucagon signaling pathway—Homo sapiens (human)	2.409	0.031
	Neuroactive ligand–receptor interaction—Homo sapiens (human)	2.109	0.031
	Nicotinate and nicotinamide metabolism—Homo sapiens (human)	2.085	0.031
	Phenylalanine metabolism—Homo sapiens (human)	2.047	0.031
	Pyruvate metabolism—Homo sapiens (human)	2.319	0.031
	Tyrosine metabolism—Homo sapiens (human)	1.933	0.037
*PECAM*	ABC transporters—Homo sapiens (human)	3.850	0.002
	Aminoacyl-tRNA biosynthesis—Homo sapiens (human)	4.702	0.000
	Central carbon metabolism in cancer—Homo sapiens (human)	2.256	0.032
	Glycine, serine and threonine metabolism—Homo sapiens (human)	2.126	0.033
	Histidine metabolism—Homo sapiens (human)	2.153	0.033
	Mineral absorption—Homo sapiens (human)	2.362	0.030
	Protein digestion and absorption—Homo sapiens (human)	4.791	0.000
	Valine, leucine and isoleucine biosynthesis—Homo sapiens (human)	2.463	0.030

The pathways identified as being over-represented in the predictors of 30-day mortality include “ABC transporters”, see Table 4 (full results Additional file 4). Those over-represented in the group related to sTM include “Alanine, aspartate and glutamate metabolism”, see Table 4 (and Additional file 5). Finally, those over-represented in the PECAM-related group include “ABC transporters”, see Table 4 (and Additional file 6), see Fig. 3a). Only one pathway, “Central carbon metabolism in cancer” was over-represented in all three sets, see Fig. 3b, highlighting the importance of energy dysregulation to both sepsis pathology and endothelial dysfunction. On the other hand, many pathways are common to PECAM and mortality, including “ABC transporters”. “Histidine metabolism” and “Valine, leucine and isoleucine metabolism”, were both found to be over-represented in both the mortality and PECAM marker sets, as shown in Fig. 4b. This once more highlights amino acids as key to the metabolic dysregulation of sepsis. Two non-metabolic pathways were identified as over-represented in the sTM markers—“Glucagon signalling” and “Neuroactive Ligand-Receptor Interaction”. These indicate some of the regulatory pathways that become dysregulated in sepsis leading to metabolic dysregulation.

## Discussion

We compared the utility of the novel metabolic and endothelial markers against current standard risk markers (septic shock diagnosis and SOFA score) to predict 30-day mortality.

The sepsis 3 guidelines identify high risk patients as having septic shock. In this study a septic shock diagnosis was only a slightly better predictor of 30-day mortality than chance, with an AUC of 0.573, see Fig. 1a. This was in line with the 46% crude mortality observed for septic shock by the Sepsis Definitions Task Force [5] and indicates that septic shock under the 2016 criteria was an ineffective predictor of 30-day sepsis mortality.

Many previous studies of sepsis survival have used the SOFA score as a predictor of sepsis mortality. With an AUC of 0.777, see Fig. 1c, the predictive ability of the SOFA score was in line with previously reported values, such as Langley et al. [20], with a predictive accuracy of SOFA score of 68% in their initial population and 61% in their predictive population and Wang et al. who found an AUC of 0.56 for their cohort for a similar study [40].

We found that patients with liver disease had a higher risk of mortality. However, due to the small number of liver patients included in this data set (*n* = 5), many of whom also had another condition this may be an artifact of the data set. Incomplete data regarding BMI prevented further follow-up of this differential feature. However, the lower BMI in the non-surviving group may indicate a frailer population at higher risk of sepsis mortality.

The endothelial health indicators PECAM and sTM have previously been shown to predict poor outcomes in sepsis [10, 11]. In this study they did so with a similar efficacy as SOFA score, AUC for endothelial health of 0.769 (95% CI 0.626–0.911) AUC 0.777 for SOFA score, see Fig. 1.

The LASSO regression selected metabolite model was extremely effective at predicting 30-day mortality in our own data set AUC of 0.93 (95% CI 0.836–0.836), see Fig. 2a. This may suggest a degree of over-fitting, likely due to the small sample size in this study.

To assess the general applicability of our models, we tested them on other published sepsis metabolomics data sets [20, 41–43] produced mixed results. Models that included all overlapping metabolites with coefficients determined by the LASSO regression, produced an AUC of better than 0.6 in two out of three data sets tested and around equal to chance in the other. However, these data sets were very different in metabolite coverage. Only measurements of 8 or 9 out of the 15 LASSO identified metabolites were available in each of the tested data sets. We then retested these data sets using only the metabolites included in each data set and finding data set-specific coefficients. This produced better outcomes, with AUCs of around 0.84 in all three cases, the ROC curve for the Wang model shown in Fig. 2b.

This suggests that while the exact patterns of metabolic changes vary between patient populations there was a consistent set of metabolites that are important to sepsis outcome. This assumption is further reinforced by the high variability seen in the measurements within each data set and the low number of individually significantly different metabolites (see supplementary data).

The consistent importance of amino acids, carnitines, fatty acids and lipids and TCA cycle derivatives as markers of sepsis outcome was also shown by Wang et al. [40] who identified isoleucine, pyruvate (also identified in this study), alanine, lactate, acetylcarnitine (related to markers identified here) and lyso-phosphytidyl cholines and glycines as consensus markers of mortality prediction across multiple metabolomics studies. The data set from Wang et al. [40] showed a 95% confidence interval range of 0,69–0,84. This model included a similar set of metabolites to our model, suggesting that these are indeed ubiquitous markers of metabolic dysregulation related to sepsis outcome. However, across four different data sets the metabolic differences between sepsis survivors and non-survivors are not always consistent [20, 41–44]. Tyrosine, butyryl and acetyl carnitines, pyruvate, lactate and alanine are consistently up-regulated in sepsis non-survivors. Myristoylcarnitine, histidine and asparagine are up-regulated in this data set but down-regulated in one other study. Isoleucine and glutamate are down-regulated in this data set but up-regulated in at least one other data set (although also down-regulated in at least one other). It is possible that this may reflect underlying differences in the populations studied, for example, the average ages of the populations and possibly underlying conditions may contribute to the metabolic states of the population.

Over representation analysis of the LASSO identified metabolites highlighted central carbon metabolism and various areas of amino acid metabolism as key to mortality biomarkers.

This analysis also showed that central carbon metabolic dysregulation was common to identifying biomarkers for 30-day mortality and the levels of two different endothelial health biomarkers, again in line with Wang’s findings. Further amino acid metabolism pathways including those of Alaine and Leucine were identified as key to the groups of metabolites correlated with PECAM and sTM concentration, again highlighting the link between endothelial dysfunction, metabolic dysfunction and mortality in sepsis patients. Thirteen metabolites correlate with both PECAM and 30-day mortality, whereas only six correlate with both sTM and mortality. This could indicate that the differing functions of PECAM and sTM contribute in different ways to the pathology of sepsis, this has previously been seen as differences in the timing of PECAM and sTM changes in plasma were associated with different risks in sepsis patients [29, 45]. Five metabolites are common to both endothelial biomarker models. The general low level of overlap between metabolites correlated with sTM and PECAM concentrations may indicate that these two markers of endothelial health act in different mechanisms—although their concentrations are quite well-correlated in this data set. PECAM being largely associated with changes to the junction between endothelial cells and interactions with platelets, while sTM can indicate more direct damage to the endothelial cells and altered inflammatory response [46, 47]. Sphingosine 1 phosphate, had a negative coefficient in all three LASSO models. Sphingosine 1 phosphate has previously been shown to be produced by endothelial cells. Furthermore, low sphingosine 1 phosphate plasma levels have been shown to have an increased risk of kidney injury and coagulation disorders in sepsis patients [48]. It is, therefore, possible that the decreased sphingosine 1 phosphate associated with endothelial damage markers and 30-day non-survival may be linked to the positive correlation of urate, potentially indicating reduced kidney function, with endothelial damage markers and 30-day non-survival. Histidine rich glycoprotein has been shown to influence sepsis survival and to be involved in the maintenance of endothelial barrier integrity [49–51], in our study we found higher levels of histidine correlated with non-survival and higher levels of endothelial damage biomarkers. It is possible that this may indicate that there was less histidine incorporated into histidine rich glycoprotein, or that it was being broken down producing more free histidine. The metabolites identified in this study, therefore, indicate not only a link to 30-day sepsis survival potential but also to possible mechanisms that mediate this outcome. However, as this was not a mechanistic study further investigation is warranted.

Previous studies have shown several broad patterns in changes to metabolites, summarised and confirmed by Wang et al*.* in 2020 [40], including that amino acid metabolism, and central energy metabolism have been identified in many studies as being altered between either sepsis patients and healthy or non-septic controls or between sepsis survivors and non-survivors [40]. These results are generally very similar to the findings from this study. The two amino acids measured by us to be lower in sepsis non-survivors, glutamate and isoleucine, have also been measured to decrease in some other studies [20]. While the amino acids, TCA cycle components and carnitines found to be increased in our study are commonly increased in several studies [20, 42, 43]. The increased representation of central energy and amino acid metabolism pathways seen in differential metabolites for sepsis survival in this data set are like the metabolic pathways of importance identified by Wang et al*.* [40].

Previously sTM and PECAM levels in patient plasma have previously been shown to be associated with poor outcomes in sepsis [10–12, 14, 52]. In this study an overlapping, set of metabolites correlates with each of these endothelial damage markers. Markers of amino acid metabolism and central energy metabolism are enriched pathways among metabolites linked to endothelial damage markers and to sepsis survival. This is a similar set of changes to those previously observed in lipopolysaccharide and interferon-stimulated endothelial cells in which loss of endothelial glycocalyx was observed [24]. Given the differences between the metabolites linked to sTM and PECAM it would be interesting to examine them in more detail in a larger cohort to fully understand their contributions. For example, the branched chain amino acids which are associated with dendritic cell function [53], are more associated with PECAM. PECAM has also been shown to be associated with dendritic cell migration [54], possibly indicating a functional link to endothelial immune function. Histidine metabolism was also associated with PECAM levels and histidine rich glycoprotein has been shown to be a good biomarker in sepsis [55]. On the other hand, arginine metabolism was associated with the levels of sTM, arginine is metabolised to nitric oxide which regulates blood pressure and has been shown to be important to sepsis outcome [56–58]. Again, it will be useful in the future to examine the functional and metabolic links highlighted here.

While this study has highlighted several interesting metabolites as potential biomarkers that may link sepsis survival and endothelial damage, and it agrees with other studies in this area, there are some weaknesses that mean that further study will be important. This study used a fairly small number of patients. Due to the small number of patients the metabolite model may have been overfitted, making it less generally applicable. We have mitigated this effect by considering the model in other published data sets, it performed less well in these data sets, but in those data sets with similar metabolite sets still performed well. A further weakness is that by considering not just survival but also endothelial markers the likelihood of chance findings is increased. Finally, this study considered a single measurement of metabolites and endothelial markers, this makes it useful for exploring clinical markers but limits the mechanistic conclusions which can be drawn. It will be interesting to follow this study with a larger cohort.

## Conclusion

Our analysis of an integrated metabolomics and endothelial biomarker data set has reinforced the findings of an increasing number of studies that amino acids, TCA cycle components and carnitines are key areas of metabolic dysfunction in sepsis. We have also found that central carbon metabolism, including TCA cycle derivatives, and amino acids particularly histidine and isoleucine are indicators of levels of endothelial dysfunction markers as well as sepsis outcome and, therefore, may benefit from more focused study.

### Supplementary Information

**Additional file 1:** Excel sheets **a** Showing the compound names, which platform (LC or GC) was used to detect them, and the internal standard used for normalisation. **b** A summary of the processed data used in the analysis.

**Additional file 2:** Barcharts showing the selection of the optimal lambda values for use in LASSO analysis for **a** Mortality, **b** sTM, **c** PECAM.

**Additional file 3:** Further detail on the demographics and clinical features of the patients **a** Patient Characteristics comparing those with 30-day survival vs 30-day all-cause mortality from ICU sepsis admission, p-values from Fishers Exact test (discrete variables) or Mann–Whitney *U* test (continuous variables) (F = Female, M = Male, BMI = Body Mass Index, SOFA = Sequential Organ Failure assessment, PECAM = Platelet Endothelial Cell Adhesion Molecule, HFNOC = High Flow Nasal Oxygen, NIV = Non-invasive ventilation, PaO2/FiO2 = ratio of arterial oxygen partial pressure (PaO2 in mmHg) to fractional inspired oxygen (FiO2)) and **b** Presence of an additional condition listed as the reason for admission to hospital concurrent with the sepsis diagnosis that precipitated the admission to the ICU comparing those with 30-day survival vs 30-day all-cause mortality from ICU sepsis admission, p-values from Fishers Exact test.

**Additional file 4:** Full results of Over Representation Analysis for Mortality.

**Additional file 5:** Full results of Over Representation Analysis for sTM.

**Additional file 6:** Full results of Over Representation Analysis for PECAM.

## Data Availability

Raw data will be available at Metabolights database study number MTBLS9323 and selected R code and processed data from the analysis available on Github https://github.com/sarahmcg1 after publication.
