# Supplementary figures and images for: LASSO regression shows histidine and sphingosine 1 phosphate are linked to both sepsis mortality and endothelial damage
Source: Eur J Med Res. 2024 Jan 20;29:71. doi: 10.1186/s40001-023-01612-7 (PMC10799523; doi:10.1186/s40001-023-01612-7)

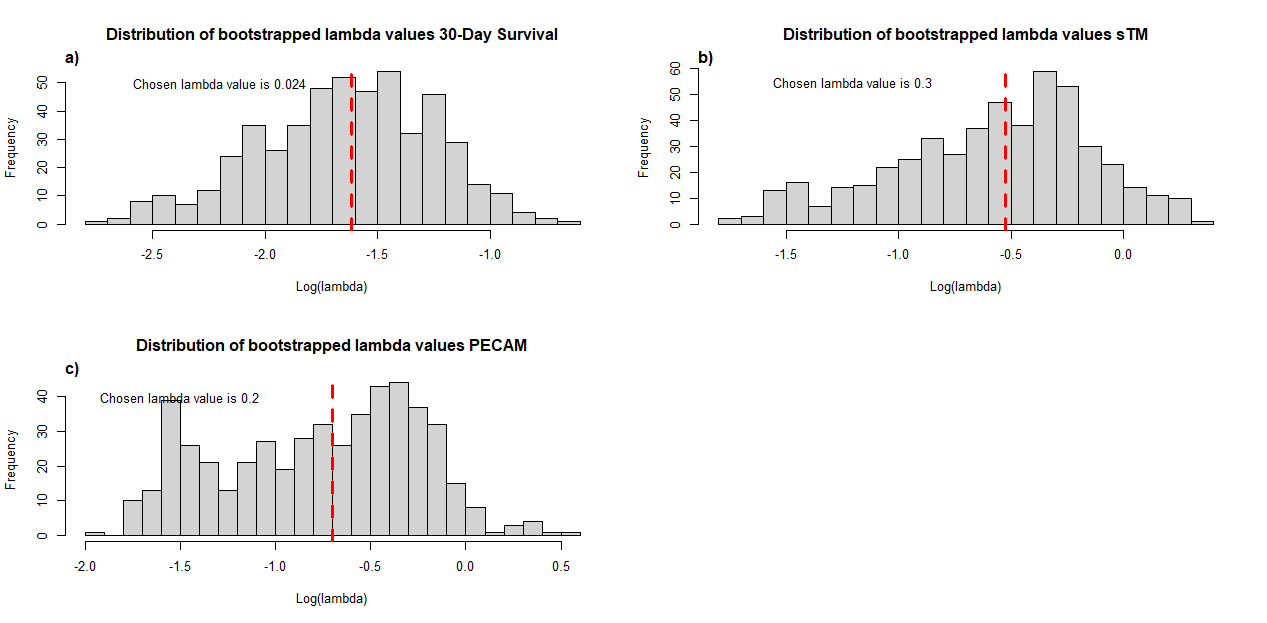

Supplement: Supplementary file 2 — Additional file 2: Barcharts showing the selection of the optimal lambda values for use in LASSO analysis for a Mortality, b sTM, c PECAM. [file 40001_2023_1612_MOESM2_ESM.png]
