# Supplementary material for: LASSO regression shows histidine and sphingosine 1 phosphate are linked to both sepsis mortality and endothelial damage
Source: Eur J Med Res. 2024 Jan 20;29:71. doi: 10.1186/s40001-023-01612-7 (PMC10799523; doi:10.1186/s40001-023-01612-7)
Supplement: Supplementary file 3 — Additional file 3: Further detail on the demographics and clinical features of the patients a Patient Characteristics comparing those with 30-day survival vs 30-day all-cause mortality from ICU sepsis admission, p-values from Fishers Exact test (discrete variables) or Mann–Whitney U test (continuous variables) (F = Female, M = Male, BMI = Body Mass Index, SOFA = Sequential Organ Failure assessment, PECAM = Platelet Endothelial Cell Adhesion Molecule, HFNOC = High Flow Nasal Oxygen, NIV = Non-invasive ventilation, PaO2/FiO2 = ratio of arterial oxygen partial pressure (PaO2 in mmHg) to fractional inspired oxygen (FiO2)) and b Presence of an additional condition listed as the reason for admission to hospital concurrent with the sepsis diagnosis that precipitated the admission to the ICU comparing those with 30-day survival vs 30-day all-cause mortality from ICU sepsis admission, p-values from Fishers Exact test. [file 40001_2023_1612_MOESM3_ESM.docx]

## *Supplemental Table 3 a) Patient Characteristics comparing those with 30-day survival vs 30-day all-cause mortality from ICU sepsis admission, p-values from Fishers Exact test (discrete variables) or Mann Whitney U test (continuous variables) (F=Female, M=Male, BMI = Body Mass Index, SOFA = Sequential Organ Failure assessment, PECAM = Platelet Endothelial Cell Adhesion Molecule,* *HFNOC = High Flow Nasal Oxygen, NIV = Non-invasive ventilation,PaO2/FiO2 = ratio of arterial oxygen partial pressure (PaO2 in mmHg) to fractional inspired oxygen (FiO2))*

|  | | **Status** | |  |
| --- | --- | --- | --- | --- |
| **Category** | **Feature** | **Survivors (Counts/Median (Lower and Upper Quartiles))** | **Non-survivors (Counts/Median (Lower and Upper Quartiles))** | **P-value** |
| *Demographic Information* | Sex | F-17,M-16 | F-10,M-9 | 1.000 |
|  | Reported Smoking Status | Ex-smoker-12,No-11,Yes-7,Missing-3 | Ex-smoker-10,No-6,Yes-2,Missing-1 | 0.679 |
|  | Age (Years) | 71(66-79) | 72(68.5-78.5) | 0.879 |
|  | ***BMI*** | ***31.419(25.703-33.951)*** | ***24.412(19.891-27.244)*** | ***0.036*** |
| *Endothelial Feature* | ***PECAM (ng/mL)*** | ***10.8(9.33-13.3)*** | ***15.4(12.25-19)*** | ***0.005*** |
|  | ***soluble Thrombomodulin (ng/mL)*** | ***13.21(10.42-18.73)*** | ***20.17(14.675-24.21)*** | ***0.006*** |
| *Measurement at ICU Admission* | ***Base Excess (mM)*** | ***1.8(1.2-2.6)*** | ***2.8(1.95-3.775)*** | ***0.020*** |
|  | Bilirubin (µM) | 213(139.4-304.5) | 188.2(113.625-248.2) | 0.211 |
|  | Creatine (µM) | 10.83(7.1-13.39) | 10.225(5.97-18.835) | 0.977 |
|  | Glucose (mM) | 108.5(102.75-111.25) | 110(102.5-112) | 0.565 |
|  | Lactate (mM) | 7(6.3-7.8) | 6.5(5.8-8.5) | 0.894 |
|  | Leukocytes (count/L) | 192(137-323) | 176(101.5-251.25) | 0.349 |
|  | Platelets (count/L) | 1.13(1.09-1.19) | 1.14(1.065-1.185) | 0.838 |
|  | ***SOFA*** | ***8(7-9)*** | ***13(10-14)*** | ***0.001*** |
|  | L of fluid given before ICU arrival | 4.11(1.6-6.625) | 2.21(1.01-4.082) | 0.201 |
|  | PaO2/FiO2 | 26.2(13.833-45.946) | 33.333(21.905-42.301) | 0.424 |
|  | Met the Criteria for Septic Shock | SepticShock-16,Sepsis-17 | SepticShock-12,Sepsis-7 | 0.391 |
|  | Ventilation Use | HFNOC-4,Invasive-13,NIV-7,No-9 | HFNOC-1,Invasive-5,NIV-4,No-9 | 0.508 |
|  | Vasopressor Use | No-12,Vasopressor-21 | No-10,Vasopressor-9 | 0.382 |
| *Medical History* | Chronic Obstructive Pulmonary Disease | Yes-5,No-28 | Yes-7,No-12 | 0.095 |
|  | Hypertension | Yes-20,No-13 | Yes-11,No-8 | 1.000 |
|  | Acute Myocardial Infarction | Yes-3,No-30 | Yes-1,No-18 | 1.000 |
|  | Stroke | Yes-4,No-29 | Yes-3,No-16 | 0.697 |
|  | Heart Failure | Yes-4,No-29 | Yes-2,No-17 | 1.000 |
|  | Chronic Kidney Disease | Yes-7,No-26 | Yes-5,No-14 | 0.739 |
|  | ***Liver Cirrhosis*** | ***Yes-0,No-33*** | ***Yes-5,No-14*** | ***0.004*** |
|  | Taking Anti-coagulant | Yes-10,No-23 | Yes-9,No-10 | 0.246 |
|  | Taking Anti-platelet drugs | Yes-8,No-25 | Yes-4,No-15 | 1.000 |
|  | Taking Immunosupressants | Yes-2,No-31 | Yes-1,No-18 | 1.000 |
|  | Cancer | Yes-3,No-30 | Yes-1,No-18 | 1.000 |
|  | ***Diabetes*** | ***Yes-3,No-30*** | ***Yes-8,No-11*** | ***0.011*** |
|  | Immune Disease | Yes-3,No-30 | Yes-1,No-18 | 1.000 |

## Supplemental Table 3 b) *Presence of an additional condition listed as the reason for admission to hospital concurrent with the sepsis diagnosis that precipitated the admission to the ICU comparing those with 30-day survival vs 30-day all-cause mortality from ICU sepsis admission, p-values from Fishers Exact test*

| **Admission Reason** | **Status at 30 days** | **Group** | **n** | **P value (Fischer)** |
| --- | --- | --- | --- | --- |
| Cardiovascular Disease | S | 0 | 13 | 0.227 |
|  |  | 1 | 20 |  |
|  | NS | 0 | 4 |  |
|  |  | 1 | 15 |  |
| Gastrointestinal Failure | S | 0 | 30 | 0.291 |
|  |  | 1 | 3 |  |
|  | NS | 0 | 19 |  |
| ***Liver*** | ***S*** | ***0*** | ***33*** | ***0.044*** |
|  | ***NS*** | ***0*** | ***16*** |  |
|  |  | ***1*** | ***3*** |  |
| Neurological | S | 0 | 29 | 1.000 |
|  |  | 1 | 4 |  |
|  | NS | 0 | 17 |  |
|  |  | 1 | 2 |  |
| Other | S | 0 | 21 | 0.771 |
|  |  | 1 | 12 |  |
|  | NS | 0 | 13 |  |
|  |  | 1 | 6 |  |
